# Free Pericardial Fat Pad Patch Repair Without Parenchymal Approximation for Severe Intraoperative Air Leak: A Technique to Optimize Limited Graft Volume

**DOI:** 10.7759/cureus.105358

**Published:** 2026-03-17

**Authors:** Tomonari Oki, Shuhei Iizuka, Yoshiro Otsuki, Toru Nakamura

**Affiliations:** 1 Department of General Thoracic Surgery, Seirei Hamamatsu General Hospital, Hamamatsu, JPN; 2 Department of Pathology, Seirei Hamamatsu General Hospital, Hamamatsu, JPN

**Keywords:** free pericardial fat pad, intraoperative air leak, patch repair, pulmonary emphysema, thoracic surgery

## Abstract

Intraoperative air leaks remain a significant challenge in thoracic surgery, particularly in patients with severe pulmonary emphysema, where conventional suture repair may exacerbate parenchymal tearing. While the use of a free pericardial fat pad (FPFP) has been reported, standard techniques typically require parenchymal approximation, which can induce tension-related injury. We describe an FPFP patch repair without parenchymal approximation, specifically designed to optimize limited graft volume. An 83-year-old male with severe bilateral emphysema underwent a left upper lobectomy for squamous cell carcinoma. During chest closure, a massive air leak became evident from an extensive pleural laceration adjacent to the hilum. Due to the fragility of the parenchyma and proximity to pulmonary vessels, direct suturing was deemed hazardous. Furthermore, as the available FPFP volume was limited because a portion had already been removed, the harvested FPFP was incised on the adipose side to expand its pleural surface area and maximize coverage. The graft was then secured as a patch over the affected area without any parenchymal approximation. The air leak was immediately resolved, and the patient’s postoperative course was uneventful. FPFP patch repair without parenchymal approximation may provide a useful option for managing severe intraoperative air leaks in fragile, emphysematous lungs. The modification to expand the graft’s surface area allows for successful repair even when available adipose tissue is limited.

## Introduction

Intraoperative air leaks are highly prevalent complications after lobectomy, with a reported incidence of approximately 58%, especially in patients with incomplete fissures or emphysema [1]. Prolonged air leaks occur in approximately 10-25% of patients and are associated with prolonged hospital stays, increased postoperative complications, and higher healthcare costs [1-4]. Conventional methods for managing air leaks include direct suturing, stapling, and the application of sheet-type sealants, such as oxidized regenerated cellulose mesh, polyglycolic acid (PGA) sheets, or collagen sponges; however, achieving complete control remains challenging, particularly in high-risk patients [5,6]. A mechanical suture line reinforced by a polydioxanone ribbon is a simple, safe, and effective method for closure of air leaks, resection, or biopsy in such cases [7]. Conversely, in emphysematous lungs or in those with large pleural defects, suture repair can generate excessive tension, leading to further parenchymal tearing. In addition, suturing can be technically challenging when pleural lacerations are located adjacent to pulmonary vessels. Although the fissureless technique, which avoids interlobar dissection [8] and stapler reinforcements [9], has been developed to prevent air leaks, patient-related risk factors frequently remain non-modifiable. Consequently, as complete prevention is challenging, it is crucial to establish surgical techniques for managing intraoperative air leaks. Recently, the use of a free pericardial fat pad (FPFP) for repairing air leaks has been reported [5,6]. However, previously reported techniques require direct tissue approximation via suture repair of the lung parenchyma, which potentially poses a risk of tension-related injury. Furthermore, the volume of harvestable FPFP is inherently limited [10], which may raise concerns regarding whether sufficient coverage can be achieved for extensive pleural defects using the available tissue. We herein report a case of successful patch repair using an FPFP without parenchymal approximation to resolve a severe intraoperative air leak caused by unexpected extensive pleural laceration of an emphysematous lung. We also describe a technique to optimize the effective use of a limited amount of harvested pericardial fat.

## Case presentation

The patient was an 83-year-old male with a heavy smoking history (Brinkman Index 1000; 50 pack-years) and bilateral emphysema with normal spirometry (Table 1).

**Table 1:** Results of preoperative pulmonary function testing. FVC: forced vital capacity; FEV1: forced expiratory volume in one second; FEV1/FVC: forced expiratory volume in one second to forced vital capacity ratio.

Parameter	Measured value	Predicted value	% of predicted
FVC (L)	3.16	3.19	99.1%
FEV1 (L)	2.05	2.21	92.8%
FEV1/FVC ratio (%)	64.8	65.7	98.6%

**Figure 1:**
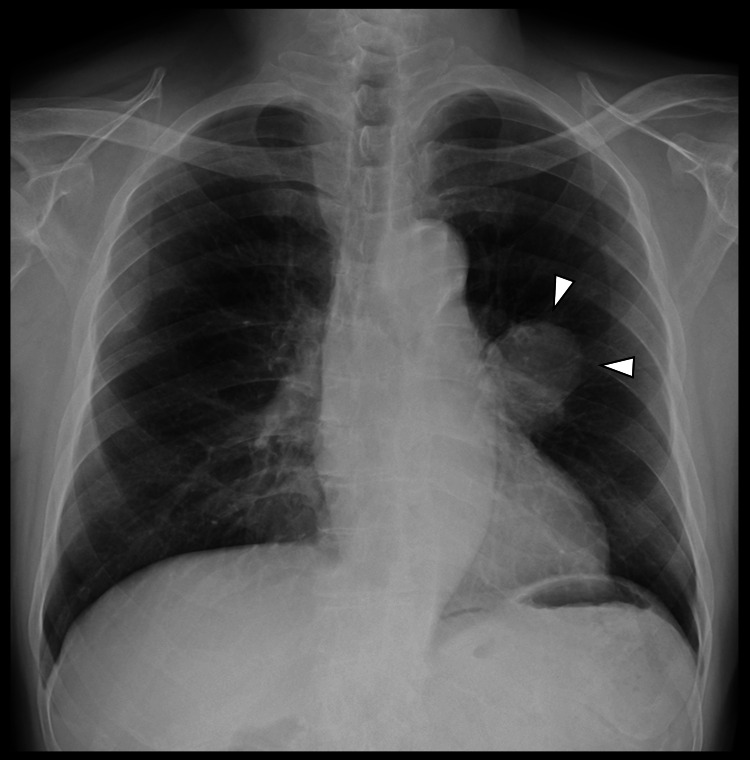
Preoperative chest radiograph. A mass is identified in the left lung (white arrowheads).

He presented with hemoptysis and coughing for six months and was referred to our hospital after a mass in the left lung was detected on a chest radiograph (Figure 1).

**Figure 2:**
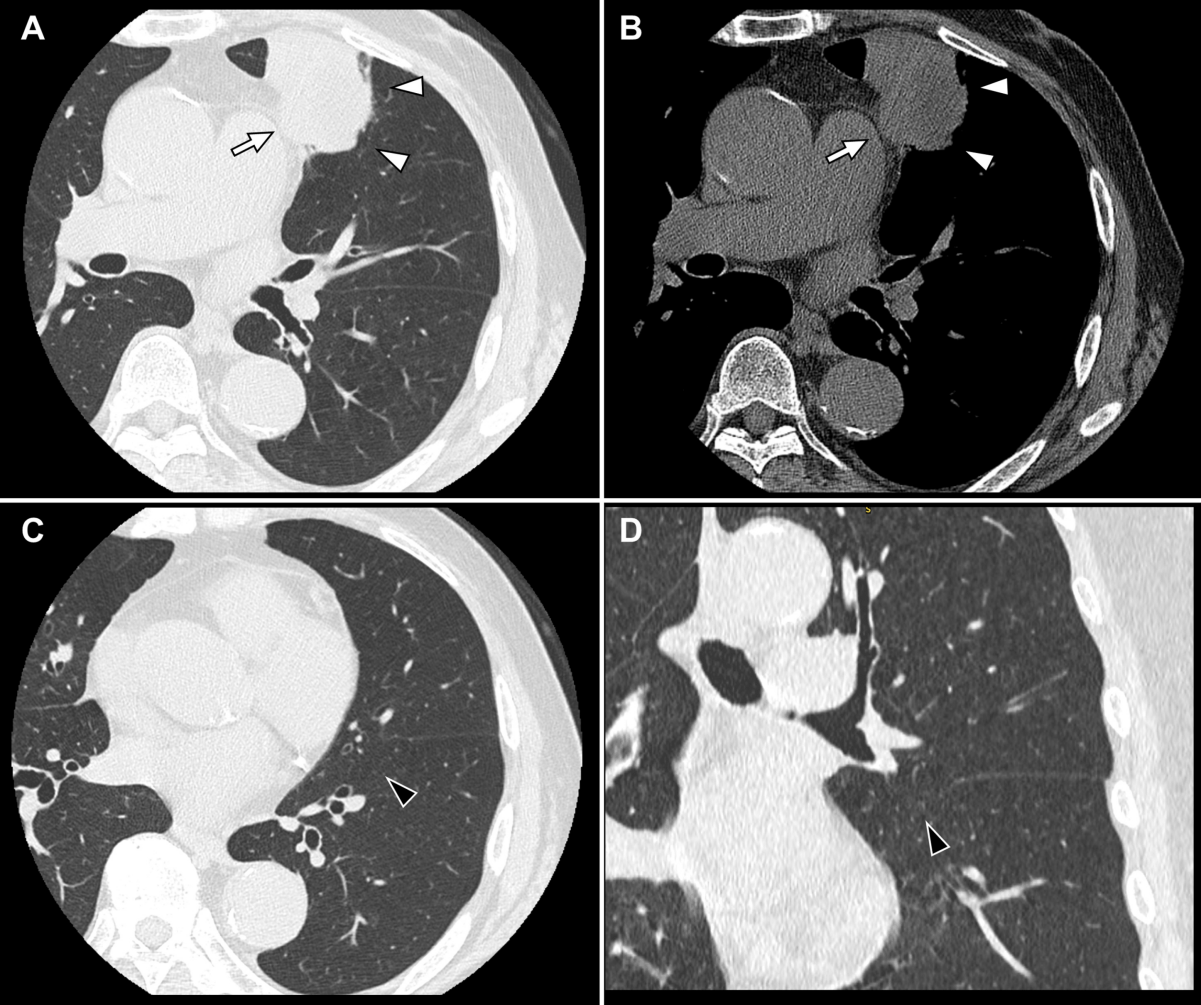
Preoperative chest computed tomography findings. Axial scans (A: lung window; B: mediastinal window) show a 52-mm mass (white arrowheads) in the left upper lobe with suspected invasion of the mediastinal fat (white arrows). An incomplete fissure (black arrowheads) is identified between the lingular and basal segments on axial (C) and coronal (D) images.

A chest computed tomography scan showed a 52-mm mass in the left upper lobe, with suspected invasion of the mediastinal fat (Figures 2A, 2B). The scan also revealed pulmonary emphysema and an incomplete fissure, particularly between the lingular and basal segments (Figures 2C, 2D).

**Figure 3:**
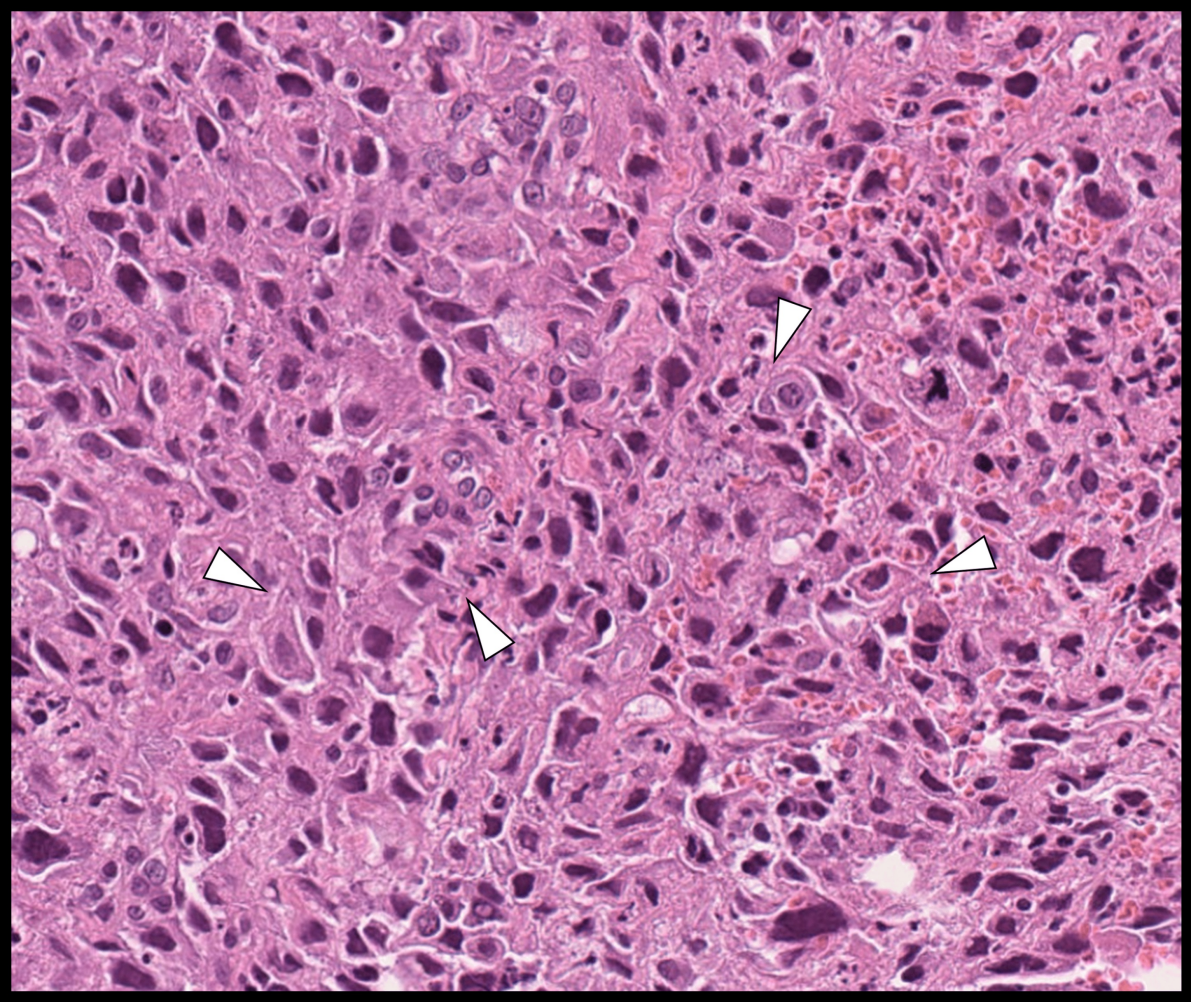
Pathological findings of the bronchoscopic biopsy. A sheet-like proliferation of atypical cells with intercellular bridges (white arrowheads) is observed, consistent with squamous cell carcinoma (hematoxylin and eosin stain, ×10).

A bronchoscopic biopsy using a 4.2-mm outer diameter bronchoscope (BF-P290; Olympus Medical Systems, Tokyo, Japan) confirmed a diagnosis of squamous cell carcinoma (Figure 3).

Molecular profiling identified no actionable genetic mutations, and the programmed death-ligand 1 tumor proportion score was 60%. A systemic workup revealed no evidence of lymph node or distant metastasis, leading to a clinical diagnosis of T4N0M0 stage IIIA. Considering the tumor's resectability, a left upper lobectomy was planned.

**Figure 4:**
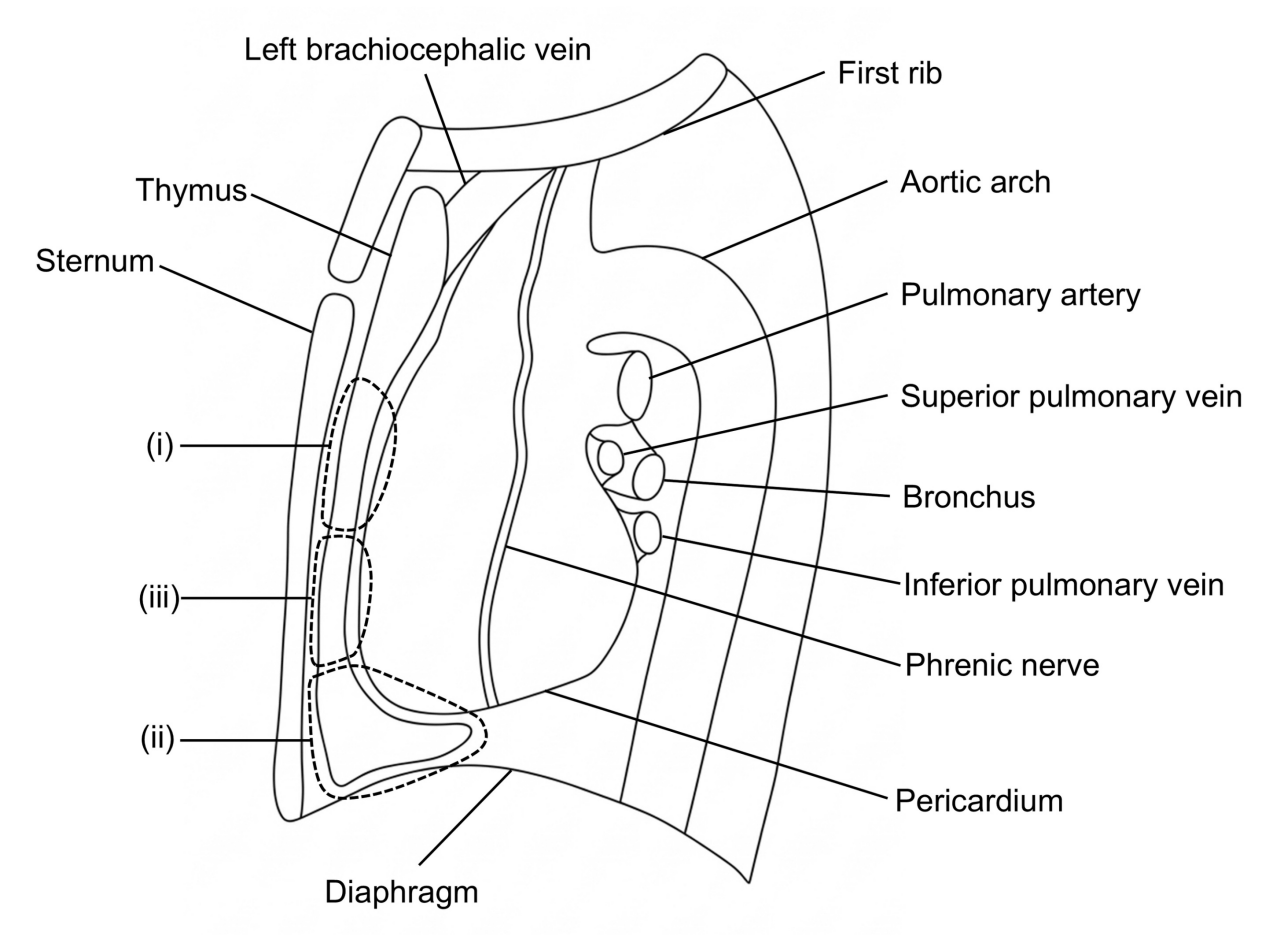
Distribution of the resected pericardial fat. Area (i) indicates the region resected en bloc with the tumor; area (ii) represents the region harvested for bronchial stump coverage; and area (iii) shows the region additionally harvested for patch repair. The illustrations were created using Microsoft Paint, version 11 and Microsoft PowerPoint 2021 (Microsoft Corporation, Redmond, Washington, USA). No artificial intelligence tools were used in the generation of these images.

A thoracotomy was performed through a 12-cm anterolateral incision via the fourth intercostal space. Severe emphysema was present, with a poorly developed and fused fissure, particularly between the lingular and basal segments. The tumor was found to invade the mediastinal fat. The tumor was resected en bloc with the involved mediastinal fat (Figure 4), while the left phrenic nerve, confirmed to be free of invasion, was preserved.

Subsequently, mediastinal lymph node dissection was performed, and the left upper lobectomy was commenced. In the standard procedure for a left upper lobectomy, the left superior pulmonary vein is typically divided first, followed by the individual pulmonary artery branches supplying the upper lobe. Caution is required during this step, as the lingular artery may originate from the basal pulmonary artery. In this conventional approach, the fissure is usually dissected to expose the pulmonary artery, and the bronchus is divided last [11]. In the present case, a fused fissure between the lingular and basal segments made it difficult to expose the lingular artery without parenchymal injury interlobarly. Therefore, we opted for a fissureless technique [8] to avoid interlobar dissection. In this approach, the superior pulmonary vein and the upper lobe bronchus were divided first, allowing for the subsequent dissection of the lingular artery from an anterior side.

Initially, the left superior pulmonary vein was divided, followed by the sequential division of the pulmonary artery branches to the apicoposterior segment. Next, the upper lobe bronchus was divided. Subsequently, the pulmonary arteries supplying the lingular and basal segments were individually exposed from an anterior side, and the lingular artery was transected. Finally, the fused fissure was divided using a stapler with buttressing reinforcement [9] to complete the left upper lobectomy. Given the patient’s history of heavy smoking and advanced-stage malignancy, the bronchial stump was reinforced with an FPFP to prevent bronchopleural fistula. The graft was harvested from the caudal end of the anterior mediastinal fat between the sternum and pericardium (Figure 4) and fixed using 4-0 monofilament sutures [12].

**Figure 5:**
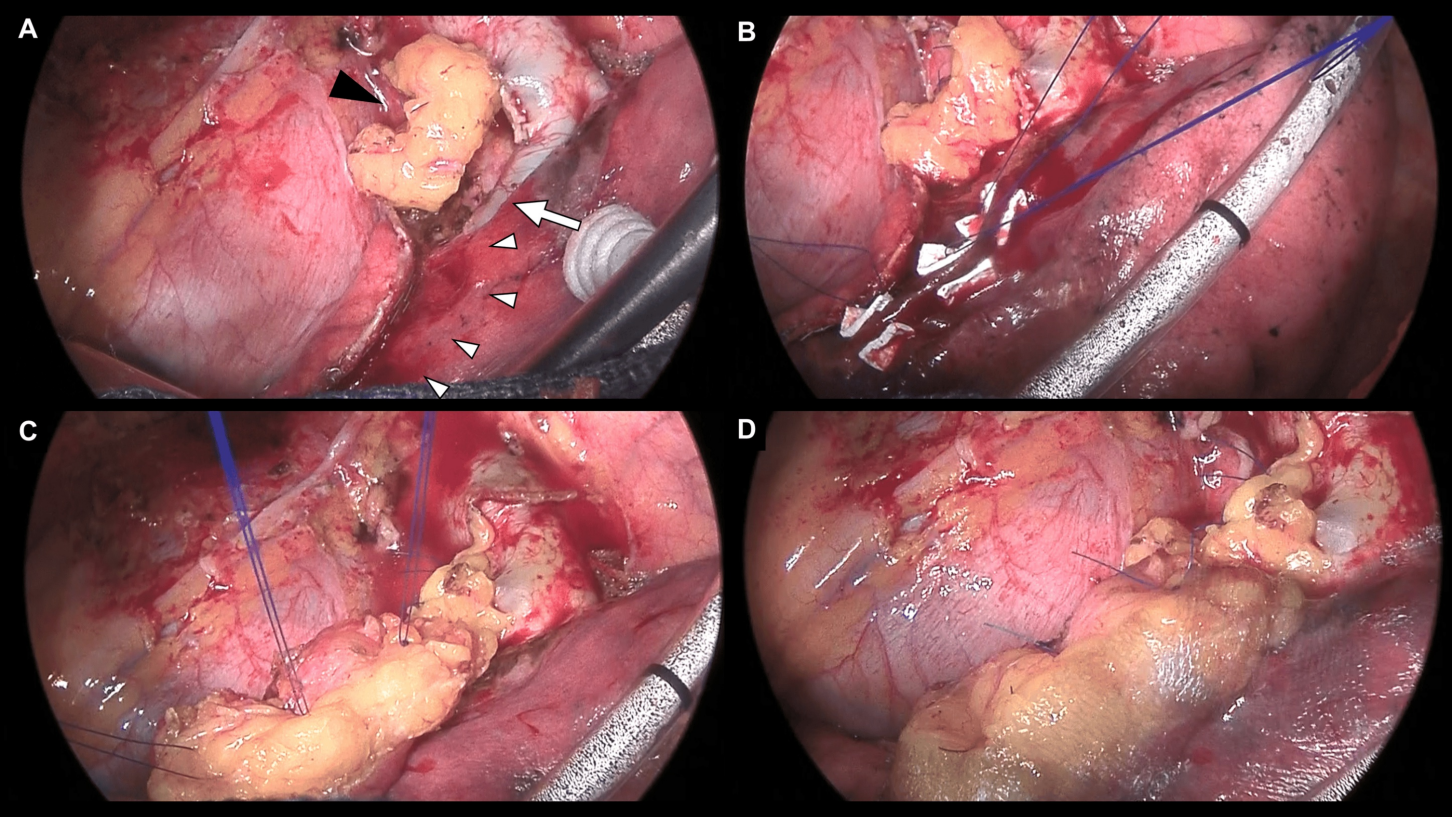
Intraoperative findings during rethoracotomy. (A) A pleural laceration measuring approximately 10 × 25 mm (white arrowheads) is present along the staple line of the basal segment. The interlobar pulmonary artery (white arrow) courses beneath the lacerated area (white arrowheads). The bronchial stump has already been reinforced with a free pericardial fat pad (FPFP) (black arrowhead). (B) Anchoring sutures are placed in the intact pleural tissue using horizontal mattress sutures with pledgets. (C) The FPFP is fixed over the defect using a parachute technique. (D) The edges of the FPFP are reinforced with polyglycolic acid sheets and fibrin glue.

A pressure-sealing test at 20 cmH2O confirmed no air leakage immediately after resection. However, upon restoring two-lung ventilation after chest closure, a massive air leak exceeding 3000 mL/min on the digital drainage system (Thopaz; Medela, Inc, Baar, Switzerland) emerged, rendering ventilation impossible. At urgent rethoracotomy, a pleural laceration measuring approximately 10 × 25 mm was identified along the staple line of the basal segment (Figure 5A).

The laceration was deemed too extensive for repair with fibrin glue or PGA sheets alone. Furthermore, suture repair was considered likely to exacerbate the laceration because of the fragility of the emphysematous parenchyma. Moreover, the interlobar pulmonary artery was located just beneath the lacerated area, rendering deep parenchymal suturing potentially hazardous due to the risk of vascular injury (Figure 5A).

Therefore, an FPFP patch repair without parenchymal approximation was employed instead of conventional repair requiring suturing. Initially, an additional FPFP graft was obtained from the area cephalad to the initial FPFP harvest site (Figure 4). However, because a portion of the adipose tissue had already been removed for tumor resection and bronchial stump reinforcement, the volume available for further harvest was limited. Given the limited volume of the harvested FPFP, we incised and spread its adipose side to enlarge the pleural surface, thereby enabling broad apposition to the defect. Next, three double-armed 4-0 monofilament horizontal mattress sutures with pledgets were placed in the intact pleura adjacent to the injury site (Figure 5B). One needle of each suture was then passed through the FPFP from the pleural side to the adipose side. The opposite needle was similarly passed through the graft and brought back to the pleural side. It was then passed through the buttressed staple line and finally returned once more through the graft to the adipose side in preparation for tying. Subsequently, the graft was parachuted down onto the pleural defect, and corresponding suture limbs were tied to secure the FPFP firmly in place (Figure 5C). Finally, fibrin glue was injected between the FPFP and the lung parenchyma, and the edges of the FPFP were reinforced with fibrin glue and PGA sheets (Figure 5D).

After confirming cessation of the air leak, the thoracotomy was closed. The total operative time, including the rethoracotomy for air leak repair, was 278 minutes. Approximately 35 minutes were devoted to addressing the air leak, including the leak test, graft harvesting, and patch closure. Intraoperative blood loss was 15 mL.

**Figure 6:**
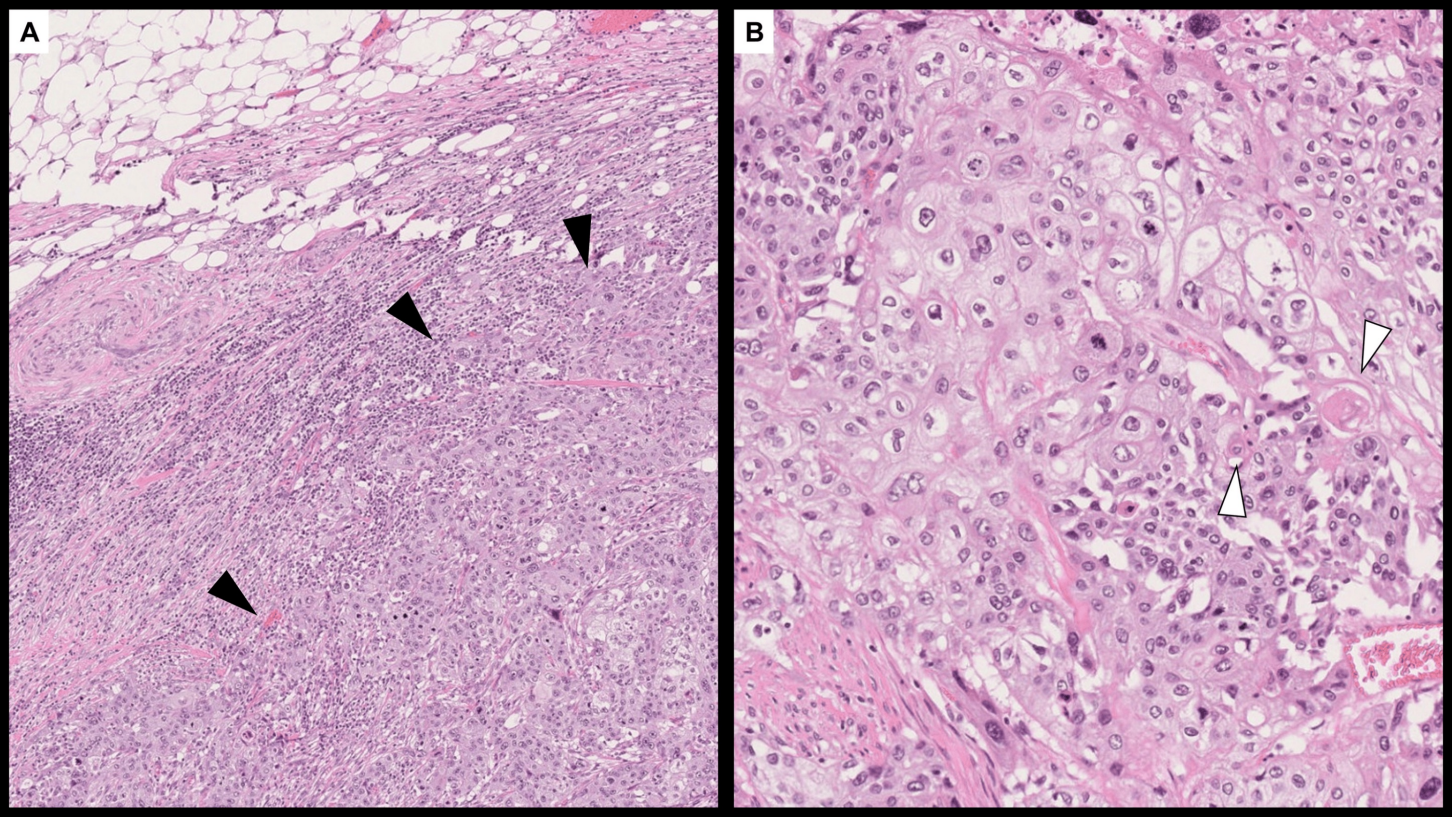
Pathological findings of the resected left upper lobe. (A) Tumor cells are seen invading the mediastinal fat (black arrowheads). (B) Sheet-like proliferation of tumor cells with keratinization (white arrowheads) is observed, confirming the diagnosis of squamous cell carcinoma. Hematoxylin and eosin stain; A: ×4, B: ×10.

Pathological examination revealed that the tumor was a 43-mm squamous cell carcinoma with invasion into mediastinal fat (Figure 6).

**Figure 7:**
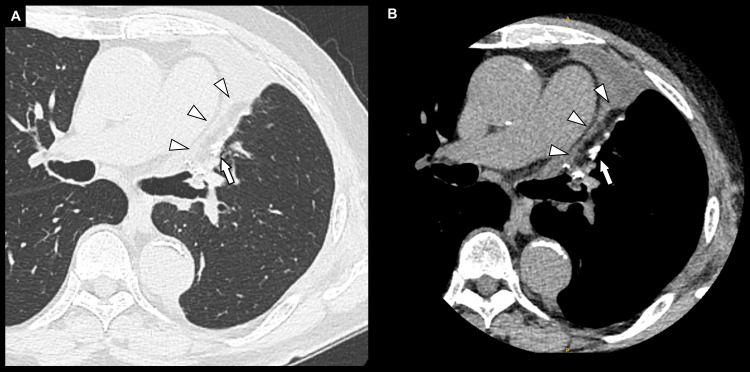
Chest computed tomography findings at eight months postoperatively. Axial views in the lung window (A) and mediastinal window (B) demonstrate favorable expansion of the left lower lobe. The anchored free pericardial fat pad (white arrowheads) is engrafted at the injury site adjacent to the staple line (white arrows).

All resection margins were negative. The final pathological diagnosis was T4N0M0, Stage IIIA. The postoperative course was uneventful, with no air leaks observed. The chest drain was removed on postoperative day (POD) 3, and the patient was discharged on POD 5. Adjuvant chemotherapy was withheld because of the patient's advanced age. A follow-up chest computed tomography scan at eight months postoperatively showed no evidence of recurrence and good expansion of the left lower lobe. The sutured FPFP was confirmed to have successfully engrafted at the injury site near the staple line of the lower lobe (Figure 7).

## Discussion

Pericardial fat, an adipose tissue layer surrounding the pericardium and covered by parietal pleura, has traditionally been used as a pedicled flap to reinforce the bronchial stump [13]. Recently, the efficacy of autologous fat grafting has gained significant attention [14]; furthermore, the use of pericardial fat as a free graft has been reported, owing to the ease and safety of its harvesting [12,15]. Previous studies have demonstrated that FPFP repair significantly shortens the duration of postoperative air leaks and chest drainage compared with conventional repair methods without an FPFP [5,6,16]. However, conventional methods using FPFPs have notable limitations.

**Figure 8:**
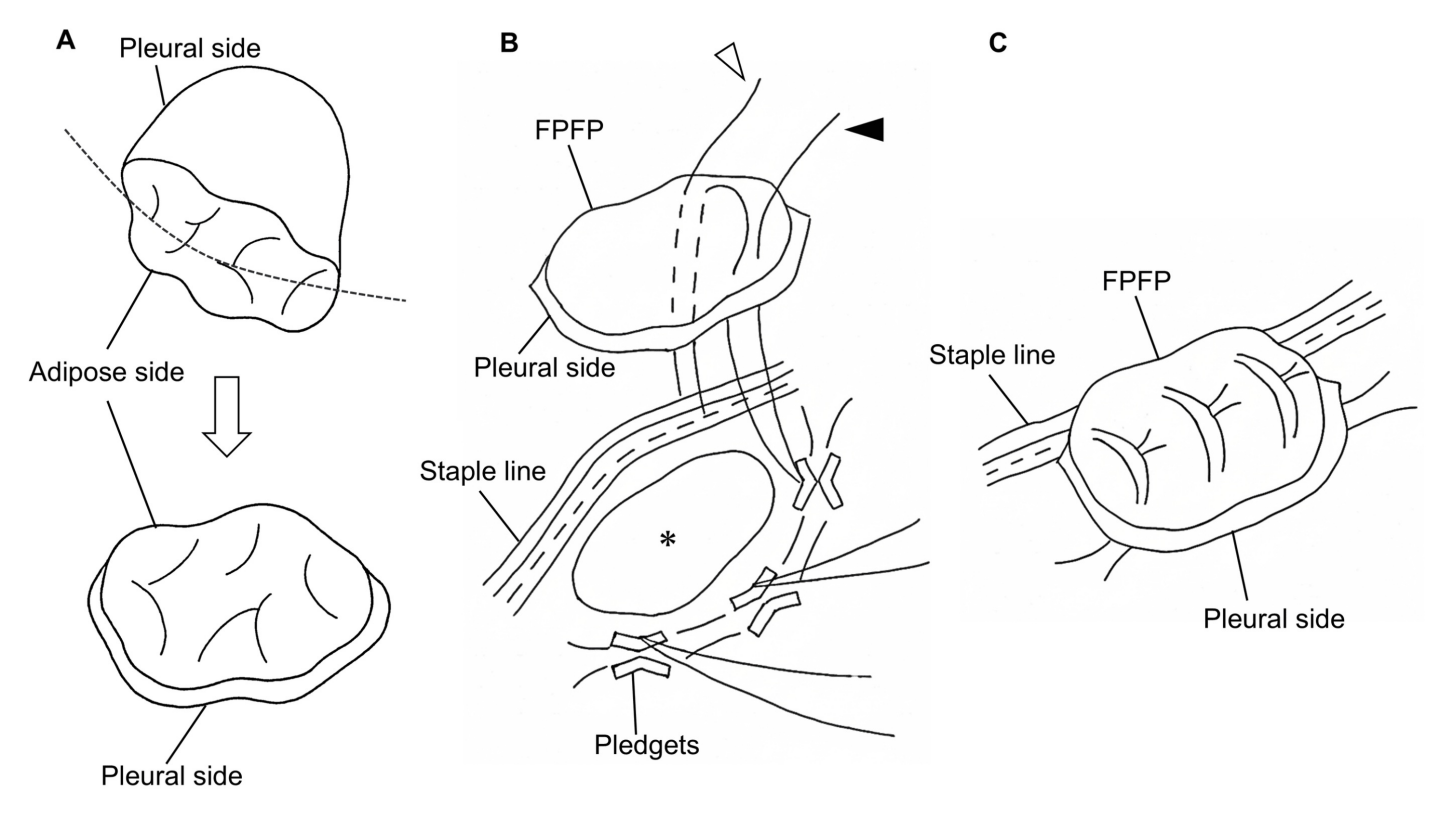
Key technical points of free pericardial fat pad (FPFP) patch repair without parenchymal approximation. (A) The adipose side of the harvested FPFP is incised (dotted line) to maximally widen the pleural surface. (B) Anchoring sutures are placed in the intact pleura surrounding the pleural defect (*) using horizontal mattress sutures with pledgets. The suture threads are passed through the FPFP from the pleural side. One limb is used to anchor the FPFP edge to the staple line (white arrowhead) and tied to the opposite limb (black arrowhead). (C) The FPFP is fixed with minimal sutures and without parenchymal approximation. The illustrations were created using Microsoft Paint, version 11 and Microsoft PowerPoint 2021 (Microsoft Corporation, Redmond, Washington, USA). No artificial intelligence tools were used in the generation of these images.

First, the harvestable volume of FPFP is inherently limited [10]. To overcome this issue, we modified the graft preparation by incising the adipose side of the harvested FPFP to expand the pleural surface (Figure 8A).

This technique maximizes the effective graft coverage area with limited graft volume, thereby facilitating both graft handling and harvesting. We consider this approach feasible even in patients with constitutionally limited mediastinal fat or when the available adipose tissue has been reduced by intraoperative surgical resection or use, as demonstrated in the present case.

Second, previous reports describe two main approaches for FPFP fixation: one in which the FPFP is used as a pledget during parenchymal suturing [5,17], and another in which the graft is applied over the defect and secured by sutures passed directly through it [6,16]. Both approaches ultimately require parenchymal approximation, potentially worsening tension-induced injury in emphysematous lungs with extensive pleural defects. In addition, they may be unsuitable for lacerations near the hilum, where deep parenchymal suturing is potentially hazardous due to the risk of vascular injury. To overcome these problems, a sutureless fat pad method using PGA sheets and fibrin glue has been reported as a fixation technique for FPFP. However, in cases with severe air leaks like the present one, its fixation strength may be inadequate for severe air leaks like the present one, posing a risk of FPFP dislocation or loss [18].

Our fixation method addresses these limitations by patch repair without parenchymal approximation. Anchoring sutures were first placed in the intact pleura adjacent to the laceration using 4-0 monofilament horizontal mattress sutures reinforced with pledgets. The sutures were directed to emerge toward the laceration side to prevent the bulk of the pledgets from interfering with graft apposition. The suture needles were then passed through the FPFP in a manner that allowed one limb to secure the graft directly, while the opposite limb was additionally anchored to the adjacent buttressed staple line (Figure 8B). The graft was subsequently parachuted down onto the defect and secured by tying the corresponding suture limbs, thereby minimizing the number of sutures required (Figure 8C).

This approach offers several advantages. First, placement of sutures in intact pleura with pledget reinforcement ensures atraumatic fixation. Second, because the lacerated pleura itself is not directly sutured, tension-related parenchymal tearing is avoided while graft migration is prevented by secure anchoring. Third, the use of shared suture limbs simplifies the procedure and reduces suture entanglement. Finally, the flexibility in selecting anchoring sites allows safe repair even near the hilum, where direct suturing would be technically hazardous.

Despite concerns regarding engraftment of free grafts, previous studies have reported a residual graft rate of approximately 62.7% at six months postoperatively [6]. Histopathological evaluations have shown preservation of adipose tissue with subsequent coverage of the visceral pleura by granulation and fibrous tissue [16,19]. Importantly, no increase in postoperative complications or infectious events has been reported [6,16]. Consistent with these findings, successful engraftment of the FPFP was confirmed by a CT scan performed eight months after surgery in the present case. The indications for FPFP extend beyond the air leak repair. This technique is also effective for preventing bronchopleural fistula following lobectomy or pneumonectomy and for reinforcing bronchial anastomoses in high-risk patients, such as those with a history of neoadjuvant therapy, heavy smoking, or diabetes [12] (Table 2).

**Table 2:** Indications for the use of a free pericardial fat pad.

Category	Clinical indications
Patient risk factors	Neoadjuvant therapy
	Heavy smoking history
	Diabetes mellitus
Surgical procedures	Reinforcement of the bronchial stump:
	–lobectomy/bilobectomy
	–pneumonectomy
	Coverage of bronchoplasty
	Repair of pulmonary air leaks

In summary, this technique offers a valuable alternative when conventional suture repair is technically demanding, particularly in severe pulmonary emphysema or extensive pleural lacerations where tissue approximation may provoke a risk of tension-induced injury. Furthermore, our technique is applicable even when only a limited volume of FPFP is available, as expansion of the adipose side creates a wider pleural surface.

## Conclusions

FPFP patch repair without parenchymal approximation may provide a useful option for managing severe intraoperative air leaks, particularly in emphysematous lungs with extensive pleural defects or perihilar lacerations where conventional suture repair is technically challenging. Expansion of the pleural surface allows efficient use of a limited FPFP volume. This approach provides a reliable and safe option for managing severe intraoperative air leaks and may be feasible at all institutions given that it has no special technical requirements.
